# Evolutionary cell biology of chromosome segregation: insights from trypanosomes

**DOI:** 10.1098/rsob.130023

**Published:** 2013-05

**Authors:** Bungo Akiyoshi, Keith Gull

**Affiliations:** Sir William Dunn School of Pathology, University of Oxford, Oxford OX1 3RE, UK

**Keywords:** chromosome segregation, kinetochores, mitosis, CENP-A, *Trypanosoma brucei*, kinetoplastids

## Abstract

Faithful transmission of genetic material is essential for the survival of all organisms. Eukaryotic chromosome segregation is driven by the kinetochore that assembles onto centromeric DNA to capture spindle microtubules and govern the movement of chromosomes. Its molecular mechanism has been actively studied in conventional model eukaryotes, such as yeasts, worms, flies and human. However, these organisms are closely related in the evolutionary time scale and it therefore remains unclear whether all eukaryotes use a similar mechanism. The evolutionary origins of the segregation apparatus also remain enigmatic. To gain insights into these questions, it is critical to perform comparative studies. Here, we review our current understanding of the mitotic mechanism in *Trypanosoma brucei*, an experimentally tractable kinetoplastid parasite that branched early in eukaryotic history. No canonical kinetochore component has been identified, and the design principle of kinetochores might be fundamentally different in kinetoplastids. Furthermore, these organisms do not appear to possess a functional spindle checkpoint that monitors kinetochore–microtubule attachments. With these unique features and the long evolutionary distance from other eukaryotes, understanding the mechanism of chromosome segregation in *T. brucei* should reveal fundamental requirements for the eukaryotic segregation machinery, and may also provide hints about the origin and evolution of the segregation apparatus.

## 2. Introduction

The numerous organisms living on Earth are divided into three domains of life (Bacteria, Archaea and Eukaryota), and transmission of genetic information from generation to generation is essential for all. Regardless of cellular organization, this requires two processes; namely, the replication and segregation of chromosomes. Compared with the DNA replication machinery, which shares several common features [1,2], the segregation machinery appears much less conserved among the three domains of life. Here, we will focus on eukaryotic segregation mechanisms and refer readers to recent reviews on prokaryotic segregation processes [3–6].

## 3. Molecular mechanism of chromosome segregation revealed from studies of popular eukaryotes

**Figure 1.**
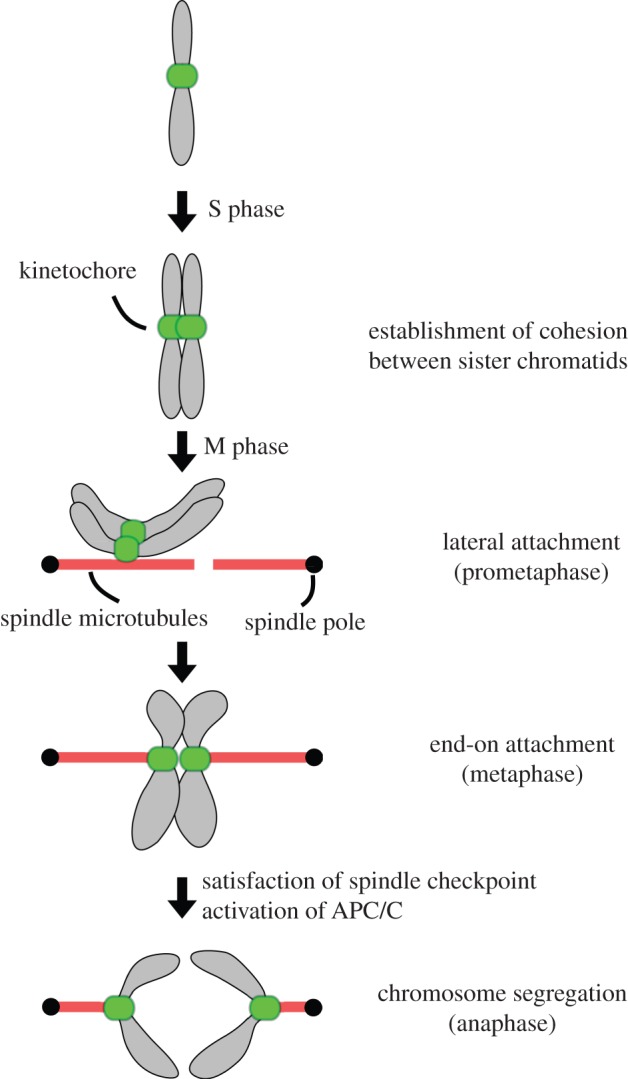
Mitotic chromosome segregation. Chromosomes are duplicated during S phase, and cohesion is established between sister chromatids. When cells enter mitosis, a bipolar spindle is assembled. Kinetochores initially form lateral attachments to spindle microtubules, which are then converted to end-on attachments. When all chromosomes form bi-oriented attachments (i.e. sister kinetochores attach to microtubules emanating from opposite poles), the spindle checkpoint is satisfied and the APC/C gets activated. This leads to the dissolution of cohesion so that the sister chromatids segregate away from each other.

During the last 40 years of research, basic mitotic mechanisms were elucidated using powerful model systems such as budding yeast, fission yeast, sea urchin, *Xenopus* egg extracts, worms, flies and mammalian tissue culture cells. The following picture has emerged from these studies (figure 1). The CDK/Cyclin complex drives cell cycle progression by promoting DNA replication and subsequent mitotic events through phosphorylation of hundreds of substrates [7–9]. Duplicated sister chromatids are held together by the cohesin complex [10,11]. This physical association enables cells to recognize which chromosomes to split during mitosis. Chromosome segregation depends on spindle microtubules and kinetochores: microtubules are dynamic polymers that consist of tubulin subunits [12,13], while a kinetochore is the macromolecular protein complex that assembles onto centromeric DNA [14,15]. During mitosis, kinetochores first form lateral attachments to microtubules, which are then converted to end-on attachments. This end-on attachment allows kinetochores to use the energy produced by the depolymerizing microtubules to move chromosomes [16–19]. Accurate chromosome segregation requires that a bipolar spindle is assembled and sister kinetochores form bi-oriented attachments to spindle microtubules emanating from opposite poles [20]. Attachment errors must be corrected to avoid mis-segregation [21]. To ensure high fidelity, cells possess a surveillance mechanism (the spindle checkpoint) that monitors the status of kinetochore–microtubule attachment and prevents cells from proceeding into anaphase in the presence of erroneous attachments [22,23]. Once all chromosomes have achieved proper bi-orientation, the spindle checkpoint is satisfied and the anaphase-promoting complex (APC/C) is activated [24–27]. This results in the activation of a protease called separase that cleaves the cohesin complex so that sister chromatids segregate away from each other [28]. The APC/C also promotes mitotic exit by degrading cyclins [29].

## 4. What does ‘conserved from yeast to human’ actually mean?

The basic mitotic machinery appears well conserved among the popular model organisms mentioned earlier. When our favourite protein is conserved in both human and yeast, we often think that ‘this protein must be universally conserved across eukaryotes because human and yeast look very different!’. Is this a valid reasoning supported by scientific evidence?

**Figure 2.**
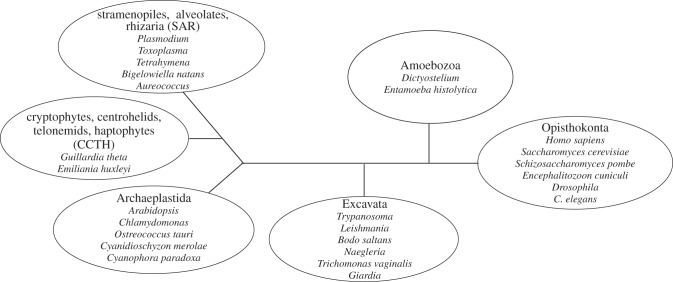
Current eukaryotic phylogenetic tree. In this unrooted tree, eukaryotes are divided into six supergroups, Opisthokonta, Amoebozoa, Excavata, Archaeplastida, SAR (stramenopiles, alveolates and rhizaria) and CCTH (cryptophytes, centrohelids, telonemids and haptophytes). Representative organisms whose draft genome sequences are available are shown as examples. The tree has been redrawn and modified from [30]. Branch lengths are arbitrary.

According to the latest molecular phylogenetic tree, eukaryotes are divided into six supergroups (figure 2) [30–32]. The popular model organisms (human, fungi, worms, flies, frogs, etc.) all belong to the supergroup Opisthokonta, which means that these organisms are closely related in the evolutionary time scale. Therefore, even if a certain protein is conserved from yeast to human, the protein may be conserved only in the Opisthokonta supergroup, not in other supergroups. It is thus essential to examine eukaryotes with a wider evolutionary distance belonging to other supergroups if we want to reveal the extent of conservation in the eukaryotic kingdom.

The availability of genome sequences now allows one to readily determine whether a protein of interest is present in distant eukaryotes using homology search programs such as BLAST [33] and HMMER [34]. A general bioinformatic assumption is that if amino acid sequences are similar, it is probably because the proteins possess a similar structure/function and a level of relatedness. However, it is important to keep in mind that a failure to detect putative homologues in fully sequenced genomes does not necessarily mean that the protein is truly absent. It is often the case that primary sequences have diverged too much to be recognized by homology search algorithms [35]. Conversely, it is also possible that even if proteins show a high level of conservation, they may function differently in different organisms owing to a different environment or other factors. For example, a highly conserved Cdc14 phosphatase plays critical roles in regulating late mitotic events in budding yeast, but not in many other eukaryotes [36]. Therefore, although bioinformatic analysis can provide a lot of information and insight, it is critical to validate the predictions experimentally.

## 5. Which evolutionarily distant organisms to study?

Understanding the extent of conservation throughout eukaryotes would be tremendously facilitated by studying the last eukaryotic common ancestor (LECA) from which all present eukaryotes diverged. However, it is not clear if such an organism exists today. As mentioned earlier, a current eukaryotic tree looks like figure 2, which is based on extensive genomic, ultrastructural and phylogenetic evidence [30–32]. The tree is unrooted because we still lack concrete views on the basal areas of eukaryotic evolution. Currently, there are several ideas proposed: rooting between unikont (Opisthokonta and Amoebozoa) and bikont (all other taxa) [37], between Opisthokonta and all other taxa [38,39], and between Archaeplastida and all other taxa [40]. In addition, based on the unique cytochrome c/c1 biogenesis [41,42], Cavalier-Smith [43] proposed that the root of the eukaryotic tree lies between Euglenozoa (or deep within the Euglenozoa tree) and all the rest of eukaryotes, which would place trypanosomes as one of the earliest branching organisms. More work is clearly needed to examine the validity and stability of these hypotheses. Regardless of the position of the root, however, it is clear that trypanosomes (Excavata) are evolutionary distant from commonly studied eukaryotes (Opisthokonta).

## 6. *Trypanosoma brucei* as a model to study chromosome segregation

We now introduce *Trypanosoma brucei* as an emerging model organism to examine the conservation/divergence of various biological processes, including chromosome segregation. Kinetoplastids are a group of unicellular flagellated eukaryotes, including parasitic trypanosomatids (e.g. *T. brucei*, *Trypanosoma cruzi* and *Leishmania* species) and free-living Bodonida (e.g. *Bodo saltans*). It is thought that the ancestor of trypanosomatids is the non-parasitic Bodonida [44–46]. *Trypanosoma brucei* is the causative agent of African sleeping sickness, which kills more than 10 000 people annually in sub-Saharan Africa [47,48], whereas *T. cruzi* and *Leishmania* species are responsible for Chagas disease and leishmaniasis, respectively. These parasites affect millions of people and animals in various parts of the world, so understanding the biology of these trypanosomatids has medical and economic relevance besides genuine scientific merits. Genome sequences are available for several species of *Trypanosoma* and *Leishmania* (from TriTrypDB; see http://tritrypdb.org) [49–53], as well as *B. saltans* (from Wellcome Trust Sanger Institute; see http://www.sanger.ac.uk) [54], which allows comparative studies among kinetoplastids to examine the evolution of parasitism as well as more generic biological questions.

### 6.1. Molecular tools

Among the kinetoplastid species, *T. brucei* is currently the most experimentally tractable organism. In addition to the genome sequence [49], many molecular tools are available (see table 1 for details). For example, efficient homologous recombination facilitates GFP-fusions for the examination of the cellular location of proteins [65], while inducible RNAi enables knockdown analysis to examine their function [75,76]. Genome-wide RNAi libraries are available [77,78,98]. Furthermore, the organism's doubling time is 6–9 h (cf. budding yeast, 2 h; fission yeast, 3 h; mammalian tissue culture, 24 h) and it thus takes only approximately 10 days to obtain clonal transfectants. Large-scale culture is also feasible [99], and one can readily perform affinity purifications (e.g. using TAP tag) to identify interacting proteins by mass spectrometry [82–86]. Although it may not be easy to arrest cells in mitosis owing to an apparent lack of the spindle checkpoint system (see below), it is possible to obtain synchronous cultures using hydroxyurea arrest and release [100] or a double elutriation method [101]. Armed with this powerful molecular toolkit, it is possible to address biological questions in *T. brucei*.

**Table 1.** Examples of molecular tools in *T. brucei.* There are at least eight drugs for selection (G418, Hygromycin, Puromycin, Phleomycin, Blasticidin, Nourseothricin/ClonNAT, Ganciclovir and FOA). Cells are typically grown in semi-defined media (SDM-79 for procyclic form [55], HMI-9 for bloodstream form [56]). Procyclic form cells readily grow up to a density of 1 × 10^7^ cells ml^−1^ (1 × 10^6^ cells ml^−1^ for bloodstream form cells) and can be frozen for long-term storage in liquid nitrogen. A subspecies, *Trypanosoma brucei brucei*, cannot infect humans owing to its sensitivity to human lytic factor [57], and is used in many research laboratories. Various monoclonal antibodies are also available [58]. Genetic exchange occurs under special circumstances (in the tsetse fly [59–61]), but it is not a widely practicable technique. Differentiation of life cycles can be reproduced *in vitro* [62–64]. GFP, green fluorescent protein; TAP, tandem affinity purification; YFP, yellow fluorescent protein.

techniques	references
epitope-tagging (e.g. TAP, FLAG, GFP and YFP) and gene deletion using homologous recombination	[65–69]
regulated gene expression using TetR and T7 RNA polymerase	[70–72]
Cre-Lox recombination	[73,74]
RNAi, genome-wide RNAi screening	[75–78]
fluorescence *in situ* hybridization	[79]
GFP tagging of chromosomes using LacO/LacI	[80,81]
affinity purification (immunoprecipitation, BioID)	[82–86]
chromatin immunoprecipitation (ChIP), ChIP-seq	[87,88]
microtubule drugs	[89–91]
live-cell imaging	[92–94]
stable isotope labelling by amino acids in cell culture	[95–97]

### 6.2. Life cycle

*Trypanosoma brucei* transmits between tsetse flies (*Glossina*) and mammalian hosts, and undergoes a complicated life cycle (reviewed in [102,103]). It proliferates in the midgut of tsetse fly as a ‘procyclic form’. After migration to the salivary glands, it develops into proliferative ‘epimastigote forms’ and then to the non-proliferative ‘metacyclic form’, which is ready to transmit into mammalian hosts. Trypanosomes are introduced into mammalian hosts upon the bite of tsetse flies. Once in the mammalian hosts, they develop into proliferative ‘bloodstream slender form’ and non-proliferative ‘stumpy form’ parasites. Once stumpy form cells are taken up by tsetse flies, they develop into the proliferative procyclic form, completing the life cycle. Each life stage is associated with unique changes in cell morphology or expressed proteins [104]. Both procyclic form and bloodstream form cells are most often used in research laboratories because they are easily cultured *in vitro*.

### 6.3. Cell structure

**Figure 3.**
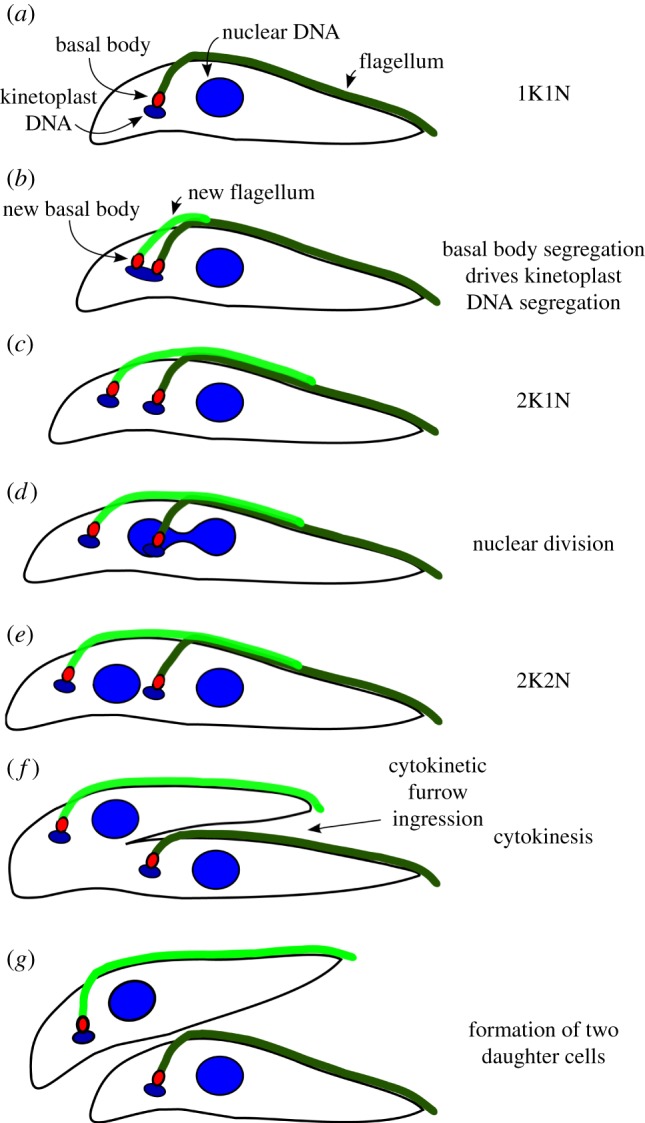
Diagram of the cell division cycle in *T. brucei* procyclic (insect) form cells. (*a*) G1 cells possess a single kinetoplast and nucleus (termed 1K1N) as well as an attached flagellum. (*b*) As the cell cycle progresses, a new basal body forms and nucleates a new flagellum. The nucleus is still in S phase when kinetoplast DNA shows an elongated morphology. (*c*) Segregation of basal bodies leads to the separation of attached kinetoplast. These cells are termed 2K1N. (*d*) Cells enter nuclear M phase, and chromosome segregation occurs. (*e*) Nuclear division is complete. These cells are termed 2K2N. (*f*) Cleavage furrow ingression occurs between the two flagella. (*g*) At the end of the cell cycle, two daughter cells are formed, and each cell inherits a single kinetoplast, nucleus and flagellum.

*Trypanosoma brucei* has a long slender shape with a single flagellum attached to the cell body (figure 3) [105,106]. The cell shape is determined by the subpellicular microtubules that underlie the plasma membrane. These microtubules are equally spaced with defined polarity (plus end in the posterior end of the cell, and minus end towards the anterior end [89]) and are highly stable owing to numerous cross-links between them [107]. This microtubule array does not disassemble during cell division. Instead, new microtubules are added between the old ones, and the array is transmitted to daughter cells in a semi-conservative manner [108]. Unlike other eukaryotes, mitochondrion and Golgi are present as single-copy organelles located at specific positions. The kinetoplast (a large structure in the mitochondrion that contains the mitochondrial DNA) is physically attached to the basal body that locates at the base of a flagellum so that the segregation of mitochondrial DNA is coupled to that of basal bodies (figure 3) [109,110]. The single Golgi is also specifically located but the physical connection to other organelles or cytoskeleton has not been determined [92,111].

### 6.4. Chromosome structure

**Figure 4.**
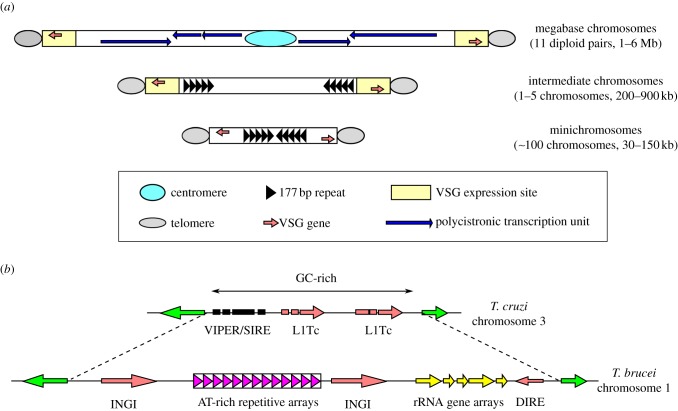
Chromosome structure and organization. (*a*) Diagram of the three different types of chromosome in *T. brucei*. Essentially, all housekeeping genes are encoded in megabase chromosomes and are expressed in polycistronic transcription units. The centromere is located in a transcriptional strand-switch region of megabase chromosomes, while such a centromere appears absent from intermediate and minichromosomes. The core of minichromosomes consists of the 177 bp repeats and is constructed in a palindromic manner with a single inversion point in the centre. The complete intermediate chromosome structure is not known, but 177 bp repeats are present. (*b*) Diagram of the centromeric region of *T. cruzi* chromosome 3 and *T. brucei* chromosome 1, based on [113–115]. Various retro-elements are found in both species (e.g. INGI, DIRE, VIPER/SIRE and L1Tc). The *T. brucei* centromere additionally contains AT-rich repeats. Ribosomal RNA gene arrays are present on a subset of chromosomes. Note that these centromeric regions retain synteny in the two species that diverged more than 200 Myr ago.

*Trypanosoma brucei* contains 11 diploid pairs of megabase chromosomes, as well as one to five intermediate chromosomes and approximately 100 minichromosomes of unknown ploidy [112] (figure 4*a*). These chromosomes are linear and have typical telomere repeats (TTAGGG) at the ends. Essentially, all the housekeeping genes are encoded in the megabase chromosomes and are transcribed as long polycistronic units with few exceptions [116–118]. The 26-Mb megabase chromosome genome contains approximately 9000 genes, including 1000 non-expressed variant surface glycoprotein (VSG) genes (most of which are pseudogenes [49]). *Trypanosoma brucei* lives extracellularly in the mammalian hosts and evades the immune response by means of antigenic variation [119]. *Trypanosoma brucei* expresses a single surface coat protein (variant surface glycoprotein, VSG) from one of approximately 15 expression sites (ESs), which locate proximal to the telomeres of megabase or intermediate chromosomes. Notably, expression of the VSGs is driven by RNA polymerase I from a special nuclear site, called the expression site body [80]. Although VSGs are highly immunogenic, *T. brucei* manages to escape the immune response by switching the expression of VSG up to once per 100 cell divisions [120–122]. This VSG switching often involves gene conversion of VSG cassettes into the active ES, creating and expressing a novel VSG gene that has not previously been seen by the immune system [123,124]. This intricate monoallelic expression and periodical switching of VSGs enable the parasites to evade the host immune response (reviewed in [125–127]), and it is thus difficult to develop effective vaccines. The parasite possesses approximately 100 minichromosomes that harbour additional VSG genes that serve as templates for recombination into one of the ESs [128]. Consistent with the concept that these small chromosomes are important for antigenic variation, they are segregated faithfully during cell division [129,130]. Minichromosomes are mostly composed of the 177 bp repeats of unknown function [131]. In addition to these linear chromosomes, circular DNA of up to 400 kb, called NR (NlaIII repeat) elements, are found in many strains, although their function remains unknown [132].

### 6.5. Centromere structure

The centromere is the chromosomal locus where kinetochores assemble to mediate the interaction with microtubules. Despite its fundamental importance, centromere structures are highly divergent and three different types are found: the regional centromere, point centromere and holocentric [133]. It is thought that regional centromeres represent the ancestral form, whereas point centromeres and holocentricity are derived features [134,135]. Determining the position of a centromere can be done in several ways. (i) By mapping the region of a given chromosome that confers mitotic and/or meiotic stability (e.g. *Saccharomyces cerevisiae* and *Arabidopsis* [136,137]). (ii) By determining the DNA sequence that associates with kinetochore/centromere proteins. For example, CENP-A (*Candida* [138]) and topoisomerase II (human [139,140] and *Plasmodium* [141]) have been used. (iii) By mapping the position of primary constrictions using a fluorescence *in situ* hybridization approach [142].

The trypanosomatid centromere was first mapped in *T. cruzi*. Kelly and co-workers [113] performed a functional mapping (telomere-associated chromosome fragmentation) and delineated the 11–16 kb GC-rich regions that confer mitotic stability (figure 4*b*). The same regions also exhibit an increased topoisomerase II activity [114], strongly suggesting that these GC-rich regions represent the centromeres in *T. cruzi*. However, this is quite unusual because centromeric DNA is comparatively AT-rich in essentially all studied eukaryotes [143]. This unusual feature might be related to the absence of CENP-A (see below). The *T. brucei* centromere was subsequently mapped based on topoisomerase II activity. Unlike *T. cruzi*, its centromere contains AT-rich repetitive arrays (20–120 kb), which are restricted to a single site on each megabase chromosome [114,115] (figure 4*b*). The unit repeat size/sequence varies among chromosomes, although some chromosomes share similar sequences; for example, chromosomes 4, 5, 8, 9, 10 and 11 possess the almost identical sequence of 147 bp (the CIR147 repeat; see below), whereas chromosome 3 has a unique 120 bp sequence [114]. Although it remains to be shown that the AT-rich repeat region confers mitotic stability and centromere activity (i.e. binding of kinetochore proteins), the fact that only one region is found per chromosome and that the region is syntenic to the *T. cruzi* centromere (although the sequence is totally different) strongly suggests that it represents a centromere in *T. brucei*. Transposable elements are found at the centromere of both trypanosomes, as in many other eukaryotes [144]. In addition, rRNA gene arrays are present adjacent to the AT-rich repeats of chromosomes 1, 2, 3, 6 and 7, although their significance is currently unknown [114].

Repetitive sequences found at the centromere of many species are thought to promote the formation of heterochromatin using endogenous RNAi pathways [145,146]. Components of the RNAi machinery (Argonaute, Dicer) are present in *T. brucei*, and small RNAs are detected from the CIR147 repeats [147,148], which are present on the centromeric region of chromosomes 4, 5, 8, 9, 10 and 11 [114]. Interestingly, small RNAs are not found from the AT-rich repeats of the other centromeres (i.e. chromosomes 1, 2, 3, 6 and 7 [148]). However, we note that these centromeres contain rRNA gene arrays (see above), which may substitute the role of CIR147 repeats. Although it remains unknown whether conventional heterochromatin is formed at the centromere, knockdown of Argonaute leads to chromosome segregation defects in *T. brucei* [149]. Notably, repetitive sequences are not found at the centromeric region in *T. cruzi* [114], an organism that does not possess a functional RNAi pathway [150].

### 6.6. Cell cycle

Similar to other eukaryotes, the cell cycle of trypanosomes consists of distinct G1, S, G2 and M phases (reviewed in [151,152]). However, as mentioned earlier, trypanosomes possess two DNA-containing organelles (kinetoplast and nucleus), both of which must be segregated faithfully. During the cell cycle of procyclic form *T. brucei*, there are distinct replication and segregation periods for kinetoplast DNA and nuclear DNA (figure 3) [153–155]. The kinetoplast finishes its DNA replication first and the kinetoplast elongation/division occurs during the S phase of nuclear DNA. The nuclear division then occurs, followed by cytokinesis that results in the formation of two daughter cells that contain one kinetoplast and one nucleus. It remains unknown how these temporal orders are established and regulated.

Trypanosomes, like many other protists and fungi [156,157], do not disassemble their nuclear envelope during mitosis (a closed mitosis [158]), and a mitotic spindle forms within the nucleus. Many eukaryotes rely on two microtubule organizing centres (MTOCs) to nucleate a bipolar spindle [159,160]. Although no distinct structure such as a centrosome or a spindle pole body is detected in *T. brucei*, electron microscopy has visualized ring-like structures, inside the nucleus and close to the nuclear membrane, that appear to nucleate spindle microtubules during mitosis [161]. It appears probable that this is a truly intranuclear MTOC specific to the spindle because *T. brucei* is one of the organisms that lack a Brr6 domain protein that appears critical to the process of nuclear envelope fenestration in spindle morphogenesis [162]. This provides yet more evidence for a set of distinct and dispersed cytoplasmic and nuclear MTOCs in *T. brucei* whose differential activation will require regulation at specific points of the cell cycle [163]. A rhomboid-shaped bipolar spindle is initially assembled and converges into two poles at opposite ends of the nucleus. Later during mitosis, this focal organization is lost and the spindle becomes bifurcated at both ends [161]. Spindle pole-specific components have not been identified thus far, and the mechanism of bipolar spindle assembly remains largely unknown in trypanosomes.

### 6.7. Conserved mitotic players

Despite the long evolutionary distance, trypanosomes do possess a reasonable proportion of the basic mitotic machinery discovered in conventional model eukaryotes. This includes the CDK/Cyclin system [164,165], cohesin complex [166,167], separase [167], condensin complex [166], Aurora B [83,168,169], APC/C [170] and proteasome [171]. Therefore, the most basic cell cycle machinery appears to be conserved in these distant eukaryotes.

Similar to all other eukaryotes, tubulins are highly conserved in trypanosomes and are essential for the segregation of both large and small chromosomes [79]. Homologues of microtubule-associated proteins are also present, including XMAP215, EB1 and CLASP, although their relevance to mitotic events remains to be investigated. Similar to other eukaryotes, Kinesin-13 (a subfamily that includes MCAK that localizes at the inner centromere [172]) plays important roles in faithful chromosome segregation [173,174]. Polo-like kinase is also present, but it does not appear to play critical roles in chromosome segregation [175–177]. Some components of the nuclear pore complex have been detected at kinetochores in metazoans [178,179], and while nuclear pore components have also been identified in *T. brucei* [180], none have been detected at trypanosomatid kinetochores thus far.

## 7. What is unique?

### 7.1. Lack of conventional kinetochores? Absence of CENP-A

One of the most striking features in kinetoplastids is the failure to identify any homologous kinetochore protein by means of extensive bioinformatic analysis [49]. Indeed, no kinetochore protein has been identified in kinetoplastids to date. It is known that kinetochore proteins show a high degree of divergence even among the Opisthokonta supergroup [181,182], and it is thus possible that the primary sequence of kinetochore proteins in kinetoplastids have diverged too much to be detectable by currently available homology search algorithms. However, this possibility seems at odds with the finding that at least a few kinetochore components are readily identifiable in various eukaryotes from all the six supergroups, including *Giardia* and *Trichomonas* [181] (B.A. & K.G. 2013, unpublished data), organisms known to have evolved at faster rates than others [183].

Furthermore, trypanosomatids do not appear to possess a centromeric histone H3 variant (called CENP-A in human), which has a conserved histone fold domain and several unique features that distinguish it from canonical histone H3 [184]. Using this criterion, CENP-A candidates are readily identifiable in all sequenced eukaryotes except kinetoplastids (*T. brucei*, *T. cruzi*, *Leishmania* and *B. saltans* [143,184]). *Trypanosoma brucei* contains four canonical histones (H2A, H2B, H3 and H4) and four histone variants (H2AZ, H2Bv, H3v and H4v), as well as divergent H1 linker histones [185–187]. It is highly unlikely that H3v is a centromeric histone H3 variant; the gene is not essential for viability, and the protein is enriched at telomeres and transcription termination sites (although it is not known whether H3v is also enriched at centromeres [87,188]). Furthermore, none of the other histone variants (H2AZ, H2Bv, H4v) or histone modifications has been associated with centromeric function to date [87,189,190]. The absence of CENP-A in all sequenced kinetoplastids strongly implies its true absence, suggesting that their kinetochores may be different in a fundamental manner. It is essential to identify kinetochore components and examine whether kinetoplastid kinetochores are completely different or share any similarity with kinetochores of other eukaryotes.

Although no kinetochore-specific component is known, some proteins exhibit putative localization to kinetochores in addition to other locations. The Aurora B kinase, a component of the evolutionarily conserved chromosomal passenger complex, shows a dynamic localization pattern during mitosis in diverse eukaryotes [191]. It initially appears on chromatin at the onset of mitosis, localizes onto kinetochores during metaphase, and then moves onto the spindle midzone and cytokinetic furrow during anaphase. A similar localization pattern was observed for TbAUK1 (one of the three Aurora kinase homologues in *T. brucei*), which shows punctate signals on metaphase chromosomes, probably representing its kinetochore localization [83]. Microtubule-severing enzymes, Spastin and Fidgetin, also show dots in the nucleus (not cell cycle regulated) and may represent their kinetochore localization [192].

### 7.2. Insufficient number of kinetochores for chromosomes?

Although kinetochore proteins have not been identified in kinetoplastids, ultrastructural studies have detected electron-dense plaques within the nucleus [158,161,193]. These plaques are visible only in mitotic cells and appear to interact with spindle microtubules (up to four in *T. brucei* [161]), suggesting that they are probably kinetochores. However, there are several peculiarities with the structure. When these putative sister kinetochore pairs interact with microtubules from opposite poles (metaphase-like state), they exhibit a back-to-back configuration without distinct space between the two structures [161]. This contrasts with other eukaryotes that have a certain distance between sister kinetochore pairs. In human, this region is called the inner centromere where cohesins and chromosomal passenger complexes are enriched to mediate cohesion between sister chromatids and to promote attachment error correction [191,194]. The apparent lack of an inner centromere region could reflect a fundamental difference in centromere/kinetochore designs in *T. brucei*, and raises questions about where/how cohesins and passenger proteins are accumulated. In addition, the number of kinetochore-like plaques detected does not match the number of chromosomes in all trypanosomatids studied to date. Only up to eight plaques were visualized in *T. brucei* [193], an organism that contains 11 homologous (i.e. 22) megabase chromosomes and approximately 100 small chromosomes. Similarly, only 10 plaques were detected in *T. cruzi* (32 chromosomes [195]), and six in *Leishmania* (36 chromosomes [196]). Although it is possible that the discrepancy derives from experimental difficulties, a similar approach has detected 14 sister kinetochore pairs in *Plasmodium falciparum* [197], an organism that has 14 chromosomes [198]. It is therefore possible that kinetochores are assembled only on a subset of chromosomes or that centromeres of multiple chromosomes may cluster together to assemble a single kinetochore in trypanosomatids. It will be necessary to identify kinetochore proteins to gain insights into this enigma. Furthermore, in *T. brucei*, there are approximately 100 small chromosomes that appear to lack centromere activity [114], while the number of spindle microtubules is fewer than 100 [161]. Although several models have been proposed [193,199], the segregation mechanism of small chromosomes remains enigmatic. It is interesting to note that a similar phenomenon is observed in *Ostreococcus tauri*, the smallest known eukaryote [200]. Cryo-electron tomographic reconstitution visualized only approximately 10 spindle microtubules (note that kinetochore plaques were not visible in this study), although this organism contains 20 chromosomes. Conventional kinetochore proteins have been identified in *O. tauri*, so it will be important to reveal whether kinetochores are formed on all chromosomes, whether clustering of multiple kinetochores occur and how kinetochores interact with spindle microtubules.

### 7.3. Absence of the spindle checkpoint?

**Figure 5.**
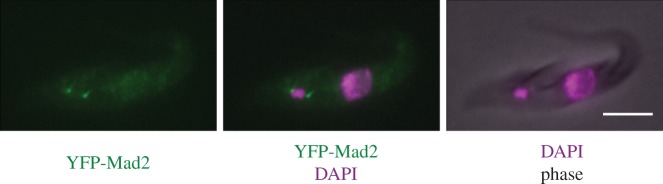
TbMad2 localizes at the basal body area. One allele of TbMad2 was endogenously tagged at the N-terminus with YFP. Similar results were obtained with C-terminally tagged Mad2. Cells were fixed with 4% formaldehyde and stained with DAPI. Scale bar, 5 µm.

The spindle checkpoint is a surveillance mechanism that monitors the status of kinetochore–microtubule attachment and delays mitotic progression until all chromosomes achieve proper bi-orientation [201]. Although some organisms do not require the spindle checkpoint for their proliferation or development under normal conditions (e.g. budding yeast, fission yeast and flies [22,23,202,203]), its presence in diverse eukaryotes indicates that it is probably critical in the wild, where quality of life is not necessarily so assured. Spindle checkpoint components include Mad1, Mad2, Mad3 (BubR1), Bub1 and Bub3 [201]. It was proposed that Mad2 plays a crucial role in amplifying the checkpoint signal by undergoing conformational changes [204]. In trypanosomatids, only Mad2 can be identified by its primary sequence and the possession of a Mad2-like HORMA domain (Tb927.3.1750/TbMad2) [205]. TbMad2 is relatively well conserved (41% identity between *T. brucei* Tb927.3.1750/TbMad2 and human Mad2, 41% between *S. cerevisiae* and human, and 36% between *T. brucei* and *S. cerevisiae*). We found, however, that YFP-tagged TbMad2 in procyclic form cells shows a constitutive localization to the basal body area (figure 5), and does not show any kinetochore or nuclear signal during normal mitosis, nor even when spindle microtubules are disrupted by microtubule drugs (B.A. & K.G. 2013, unpublished data). Furthermore, a well-conserved Mad2-binding motif [206] is not present in the TbCdc20 protein, a critical target of the spindle checkpoint pathway in other eukaryotes. These observations suggest that TbMad2 is unlikely to be a functional homologue of the spindle checkpoint Mad2 protein despite the high level of sequence similarity.

Consistent with this possibility, there is no strong evidence that trypanosomatids possess a functional spindle checkpoint. Perturbation of spindle assembly does not prevent cells from undergoing cytokinesis [207], although the exact cell cycle state of the nucleus was not examined in this study. We therefore monitored the localization of AUK1 (an Aurora B homologue that shows dynamic localization patterns during mitosis), but did not obtain any evidence that cells are arrested in a pre-anaphase state in response to spindle damages (B.A. & K.G. 2013, unpublished data). Furthermore, inhibition of nuclear DNA replication prevents nuclear division, but cytokinesis still occurs [207]. These results suggest that cytokinesis occurs regardless of the state of nuclear DNA or bipolar spindle formation. This raises an important question: how are the cell cycle controls operating in this organism? One suggestion (as alluded to above) is that cells may monitor the state of basal bodies rather than nuclear DNA [207], an interesting possibility in the light of the evolutionary history of flagellated eukaryotes. It is thought that LECA possessed flagella and basal bodies in addition to the nucleus [208,209]. Because trypanosomes might be one of the earliest branching eukaryotes [43], it is interesting to speculate that an ancient function of the spindle checkpoint component Mad2 might have been to monitor the segregation of basal bodies/flagella, consistent with the TbMad2's localization to the basal body area (figure 5). Future studies are needed to reveal the function of Mad2 in trypanosomatids, which might provide hints about the origin of the spindle checkpoint system.

## 8. Perspectives: evolutionary cell biology

Studies in powerful model eukaryotes have led to an in-depth understanding of the mechanism of biological processes. Although it is essential to continue these efforts, it is also important to perform comparative studies to understand the extent of conservation/divergence across eukaryotes. This approach, termed ‘evolutionary cell biology’ [210], also aims to understand the design and working principles of fundamental biological processes, as well as to reveal their evolutionary history (e.g. centrioles/cilia/flagella [209], nucleus [211,212], cytoskeleton [213,214] and mitosis [215–218]). The goal of chromosome segregation is the partition of duplicated chromosomes. If there is a completely different way of achieving this task, understanding such a mechanism could provide insights about fundamental requirements for the process. Furthermore, if we are to obtain a complete understanding of the segregation machinery, we need to understand where it came from and how it evolved. Studying evolutionarily distant organisms is one way to obtain hints about the evolution of biological processes.

Here, we have focused mainly on the structure at the centre of the segregation mechanism (i.e. the kinetochore), but a lot of other mitotic processes deserve to be investigated as well. For example, the molecular mechanism of bipolar spindle assembly and cytokinesis remains obscure in trypanosomatids [219–221]. Furthermore, cells must coordinate various events in space and time. In *T. brucei*, mitochondrial DNA replication is achieved prior to the completion of nuclear DNA, but the molecular mechanism that facilitates this temporal periodic order is not known. Interestingly, in *Cyanidioschyzon merolae* (a red alga), the DNA replication of plastids and mitochondria also precedes that of the nucleus [222]. Future studies should reveal if similar regulatory principles operate in trypanosomatids. Regulating the position of the nucleus and other organelles relative to the site of the cytokinetic furrow is also critical to allow the accurate partition of segregated chromosomes [223,224]. Differential positioning of the cytokinetic furrow occurs in different life stages, although little is known about the molecular mechanism [225]. By addressing these questions, we should obtain better understanding of the mitotic mechanism in this distant eukaryotic parasite. Because *T. brucei* causes devastating African sleeping sickness disease, understanding its mechanism of chromosome segregation and the difference from the mechanism used by other organisms may also facilitate drug target identification, and therefore have great relevance for human and animal health.

## 9. Acknowledgements

We thank Robin Allshire and Andrea Musacchio for fruitful discussions. B.A. was supported by postdoctoral fellowships from the EMBO and Human Frontier Science Program. Research in the Gull laboratory is supported by the Wellcome Trust and BBSRC.
